# Effects of Left-Displaced Abomasum on the Rumen Microbiota of Dairy Cows

**DOI:** 10.3390/microorganisms14010030

**Published:** 2025-12-22

**Authors:** Zihang Qin, Yawei Sun, Jiaqi Zhang, Yunle Cui, Haiyang Hu, Yuefeng Chu, Xin Li, Xuelian Ma, Gang Yao, Chuanjun Wang, Bao Wang, Qiang Fu, Qi Zhong, Na Li

**Affiliations:** 1 College of Veterinary Medicine, Xinjiang Agricultural University, Urumqi 830052, China; 320232860@stu.xjau.edu.cn (Z.Q.); syw2008@xjau.edu.cn (Y.S.); 14799296902@163.com (J.Z.); mmmdmmm404@outlook.com (H.H.); 320220061@xjau.edu.cn (X.L.); maxuelian@xjau.edu.cn (X.M.); yg@xjau.edu.cn (G.Y.); fq198505@gmail.com (Q.F.); 2 Xinjiang Key Laboratory of New Drug Research and Development for Herbivorous Animals, Urumqi 830052, China; 3 Chuangjin Animal Husbandry, Yili 835213, China; 13150300967@163.com (Y.C.); 18160566323@163.com (C.W.); wb13394994888@163.com (B.W.); 4 College of Veterinary Medicine, Lanzhou University, Lanzhou 730000, China; chuyf@lzu.edu.cn; 5 Center of Diagnosis and Control for Animal Diseases in Aletai Prefecture, Aletai 836500, China; 6 Institute of Veterinary Research, Xinjiang Academy of Animal Sciences, Urumqi 830011, China; 13932533005@163.com

**Keywords:** Holstein cow, left displaced abomasum, rumen microbiota, 16S rRNA sequencing

## Abstract

In this experiment, the rumen microbiota of healthy cows, cows with left-displaced abomasum (LDA), and cows after 7 days of LDA treatment (LDA_7) were compared to explore the correlation between LDA and the rumen microbiota and identify early warning markers for LDA. Compared with the healthy group and the LDA_7 group, the LDA group exhibited fewer Operational Taxonomic Units (LDA: 21,361; Healthy: 23,231; LDA_7: 23,956; *p* < 0.05). The results revealed significant differences in community structure between LDA-affected cows and healthy cows, as well as between treated and untreated LDA-affected cows. Specifically, the relative abundance of *Bacteroidota* significantly increased and the relative abundance of Firmicutes significantly decreased in LDA-affected cows (*p* < 0.05). At the genus level, the abundances of *UCG_002*, *UCG_010*, and *Bacteroidales_BS11_gut_group* significantly increased (*p* < 0.01), whereas those of *Trichosporon*, *Chryseomicrobium*, and *[Ruminococcus]_gauvreauii_group* significantly decreased in LDA-affected cows (*p* < 0.01). Functional prediction analysis indicated that amino acid biosynthesis and glycerol ester metabolism were significantly downregulated in LDA-affected cows (*p* < 0.01). After treatment, the abundance of key microbial taxa (*UCG_002* and *Lachnospira*) and metabolic functions normalized to levels similar to healthy cows (*p* > 0.05). These findings suggest that changes in rumen microbiota richness and diversity could serve as early warning markers for LDA detection.

## 1. Introduction

Displaced abomasum (DA) is a prevalent nutritional metabolic disorder in dairy cows and is classified primarily as left-displaced abomasum (LDA) or right-displaced abomasum (RDA) [1]. Previous studies have indicated that the incidence of LDA exceeds that of RDA, constituting approximately 75% of DA cases [2]. The peak incidence period of LDA extends from 2 weeks prior to calving to 2~4 weeks post-partum, during which the abomasum shifts from its normal position into the left abdominal cavity and becomes positioned between the left abdominal wall and the rumen [3,4,5]. Previous studies have demonstrated that dairy cows afflicted with LDA experience a daily milk yield reduction of 8 kg when compared to cows that have not been affected by LDA [6]. LDA adversely affects the digestive system and production performance of dairy cows and can result in mortality in severe cases, leading to significant economic losses in dairy farming [7].

LDA requires prompt treatment once diagnosed to minimize its negative effects. Common treatments include medication, rolling and repositioning, and surgery [8]. Surgical treatment is frequently employed on large-scale ranches to address this condition [8]. The advantage of surgical treatment lies in the rapid repositioning of the abomasum, while the complexity of this strategy constitutes a significant disadvantage [7,8]. Surgical intervention demands a high level of operator skill and is associated with substantial costs [9,10,11]. The incidence of LDA in high-yielding dairy cows ranges from 3.0~5.0% in North America [12,13]. In Europe, it varies from 1.6~3.0%, with individual farms reporting a maximum of 18% [14]. In 2024, the incidence of LDA in dairy cows in China ranged from 1.5~5.0%, with the incidence in the Xinjiang region ranging from 1.2~4.8% [15]. At present, large-scale farms face challenges in monitoring cows for LDA at an early stage. Early diagnosis of LDA has emerged as a considerable challenge for Xinjiang’s animal husbandry industry.

The rumen is a specialized digestive organ in ruminants [16]. Its proper function ensures animal health and increases production efficiency [17]. It is the largest compartment among the four stomach chambers of ruminants, accounting for approximately 70% of the total stomach capacity, and serves as the primary site for microbial fermentation and nutrient transformation [16]. The abomasum, located to the right and below the rumen, is responsible for chemical digestion and further breakdown of proteins in the feed. The rumen microbiota functions to promote fiber degradation, convert carbohydrates into volatile fatty acids, and synthesize microbial proteins, which are essential for dairy cows to maintain energy balance and productive performance [18]. Previous results have indicated that the richness and structure of the rumen microbiota are closely correlated with digestive function, production performance, and the incidence of diseases in dairy cows [16]. Hence, an interaction likely exists between the rumen microbiota and LDA. LDA can compress the rumen, leading to a displacement of the rumen’s anatomical position, which in turn alters the rumen’s filling and motility [19]. These alterations can affect the structure and function of the rumen microbiota, disrupt the rumen fermentation environment, change the rumen pH, and impact the growth and metabolic activities of certain microbes, thereby further exacerbating the dysfunction of the rumen [20]. LDA potentially induces an imbalance in the rumen microbiota that could adversely affect digestion and nutrient absorption in ruminants [19,21]. Furthermore, an imbalance in the rumen microbiota could lead to additional digestive system diseases [22]. Therefore, regulating the balance of the rumen microbiota is beneficial for understanding the physiological mechanisms of ruminant digestion, preventing and treating LDA, improving animal production performance, and reducing the economic losses associated with animal diseases in the breeding industry. This approach is a feasible strategy for increasing cow productivity. In this study, the diversity, differences, and metabolic functions of the rumen microbiota of healthy cows, cows with LDA, and cows with successfully treated LDA 7 days post-surgery were analyzed using 16S rRNA sequencing, aiming to identify which rumen bacterial genera are altered during LDA, to elucidate the metabolic functions these genera mediate, and to determine whether changes in their abundance contribute to LDA, thereby establishing microbial biomarkers for the early detection of LDA in dairy cows.

## 2. Materials and Methods

### 2.1. Animals

Thirty cows of similar age, litter size, and days of lactation were selected from a large-scale farm in the Xinjiang region between May and July 2023; all the cows with LDA developed the condition during this period. They were divided into 3 groups: 15 healthy cows (healthy group), 15 cows with left-displaced abomasum (LDA group), and 15 cows with left-displaced abomasum 7 d after surgical treatment (LDA_7 group) (Table 1). The 15 cows in the LDA group underwent surgical treatment immediately after being diagnosed with LDA. The cows that had surgery were assigned to the LDA_7 group, and these cows were resampled 7 days post-surgery. The three groups of cows had the same rearing conditions and rearing environments; the diet consisted of a mixture of corn, silage, alfalfa hay, soybean meal, and vitamin premix.

The clinical symptoms of left-displaced abomasum in dairy cows mainly include decreased appetite, reduced rumination, weakened rumen motility, dry and scant feces, decreased milk production, reduced milk fat percentage, acetone odor in breath, palpable distended abomasum under the left rib arch, “ping” sound on percussion, possible protrusion of the left posterior ribs, inability to palpate the rumen in the left flank region, and possible mild dehydration and tachycardia [23]. Healthy cows were characterized by the absence of malodorous or purulent uterine discharge, a lack of clinical signs, and minimal variations in milk production within a one-week period [24].

### 2.2. Surgical Treatment of LDA in Cows

The surgical treatment was performed following the method of Newman [25]. Cows underwent surgery on the day of the diagnosis of LDA, during which a right paramedian incision and pyloric fixation were performed. The procedure was as follows [25]: the cow was kept in a standing position and held in place. A vertical incision was made, and a veterinary ventilator 20216241 (Yuqiu Medical Equipment Co., Ltd., Danyang, China) was inserted into the abdominal cavity with the operator’s left hand to puncture the upper part of the abomasum and expel the gas. The abomasum naturally fell back after venting and was then pulled down to the incision site. The pylorus was fixed to the area of the greater omentum by sutures. After the above steps were completed, the abdominal wall was sutured, and the abdominal cavity was closed. Nodal sutures were applied to the skin, and the sutures were removed one week after the operation [25,26]. The 15 cows with LDA received standard postoperative care after surgery, including monitoring of vital signs and provision of nutritional support, and no antibiotics were administered postoperatively.

### 2.3. Sample Collection

The rumen fluid was collected following the method of Camila [27]. Gastric catheters were used to collect the rumen contents from the three groups of cows. The veterinary gastrotube 2020047 (Yuqiu Medical Equipment Co., Ltd., China) was inserted into the middle of the rumen floor through the cow’s mouth and esophagus. To avoid contamination of the rumen samples by oral bacteria and saliva, the gastric tube was flushed with sterile saline before use. Afterward, 30~40 mL of rumen contents was withdrawn from each cow, filtered through 4 layers of gauze and stored in 5 mL centrifuge tubes. The samples were stored in liquid nitrogen, snap-frozen, and sent to the laboratory for 16S rRNA sequencing and analysis [27].

### 2.4. 16S rRNA Amplicon Sequencing of Rumen Contents

Genomic DNA was extracted from the 45 collected samples. DNA was extracted using the FastPure DNA Isolation Kit (Magnetic bead) (MJYH, Shanghai, China). After DNA extraction, the concentration and purity of the DNA were measured using a NanoDrop 2000 spectrophotometer (Thermo Fisher Scientific, Waltham, MA, USA), and the integrity of the DNA was assessed by 1% agarose (Thermo Fisher Scientific, USA) gel electrophoresis. After a sample passed the DNA quality assessment, an appropriate volume of the sample was diluted to 1 ng/µL with sterile water. The V3-V4 region was amplified via PCR with the universal primers 341F (5′-CGTAYGCGRBGCATCAC-3′) and 806R (5′-GGACTAGHNGGCTATCTAAT-3′). Aliquots were mixed according to the concentration of the PCR products, ensuring thorough mixing. The PCR products were subsequently separated by electrophoresis on a 2% agarose (Thermo Fisher Scientific, USA) gel in 1× TAE, after which the target bands were recovered. Following purification, libraries were constructed using the NEXTFLEX Rapid DNA-Seq Kit (Bioo Scientific, Austin, TX, USA), and the libraries were then subjected to quantification by an Applied Biosystems™ 7500 qPCR system (Thermo Fisher Scientific Company, USA) and analysis in Fragment Analyzer 5400 (Agilent Technologies, Beijing, China). After a sample passed the quality control tests, sequencing was conducted on an Illumina NovaSeq (Illumina Company, San Diego, CA, USA).

### 2.5. Data Processing and Statistical Analysis

The DADA2 plugin (v1.22.0) in QIIME2 (v2022.2) was utilized to filter, denoise, and merge raw sequences from all samples and to remove chimeric sequences, generating Operational Taxonomic Units (OTUs) [28]. Representative OTU sequences were compared with the Greengenes2 database (v2022.10) using the feature-classifier classify-sklearn method in QIIME (v2022.2) to obtain taxonomic annotation information [29]. OTUs annotated as chloroplasts, mitochondria, or unclassifiable to the kingdom level, along with their sequences, were removed. The proportion of sequence numbers at six taxonomic levels (Kingdom, Phylum, Class, Order, Family, Genus) was calculated based on OTU abundance and annotation information to evaluate taxonomic resolution [30]. Venn diagrams were constructed to analyze unique and shared OTUs among sample groups and count shared or exclusive OTUs. Alpha and Beta diversity analyses were conducted using the QIIME (v2022.2) diversity plugin [31]. The observed_features index assessed species diversity, while the Chao1 index reflected species richness. The Kruskal–Wallis test compared Alpha diversity indices among groups and generated plots. Bray–Curtis and unweighted UniFrac distances were calculated to evaluate Beta diversity [31]. Principal Coordinate Analysis (PCoA) was performed, and principal coordinates with the highest contribution rates were selected for plotting. Permutational Multivariate Analysis of Variance (PERMANOVA) compared microbial community structures among groups. The Kruskal–Wallis test was used for LEfSe (v1.0.8) analysis on characteristic genera (LDA score > 3.0) [32]. The Diamond tool (v2.0.13) aligned species gene sequences against the KEGG database (BLASTP e-value = 1 × 10^−5^) to obtain KEGG functions [33]. The Spearman correlation coefficient calculated correlations between differentially abundant genera and KEGG Level 3 metabolic pathways, with heatmaps generated using the pheatmap (v1.0.12) package in R [34].

The significance of differences between multiple groups was analyzed by one-way ANOVA using GraphPad Prism (v10.1.12) to assess species-level differences in the rumen microbiota and differences in the metabolic functions of tertiary pathways among the three groups of dairy cows, and the results were plotted. All statistical analyses were performed under a completely randomized design. The results are expressed as the mean ± standard deviation values; * indicates a significant difference at *p* < 0.05.

### 2.6. Ethics Statement

The study was conducted in strict accordance with the guidelines for the care and use of experimental animals as promulgated by the Animal Welfare and Ethics Committee of Xinjiang Agricultural University. The experimental protocol was reviewed and approved by the Animal Welfare and Ethics Committee of Xinjiang Agricultural University (No. 2023054). All efforts were made to minimize the suffering of the animals.

## 3. Results

### 3.1. Analysis of Bacterial Diversity

#### 3.1.1. Comparison of OTUs

**Figure 1:**
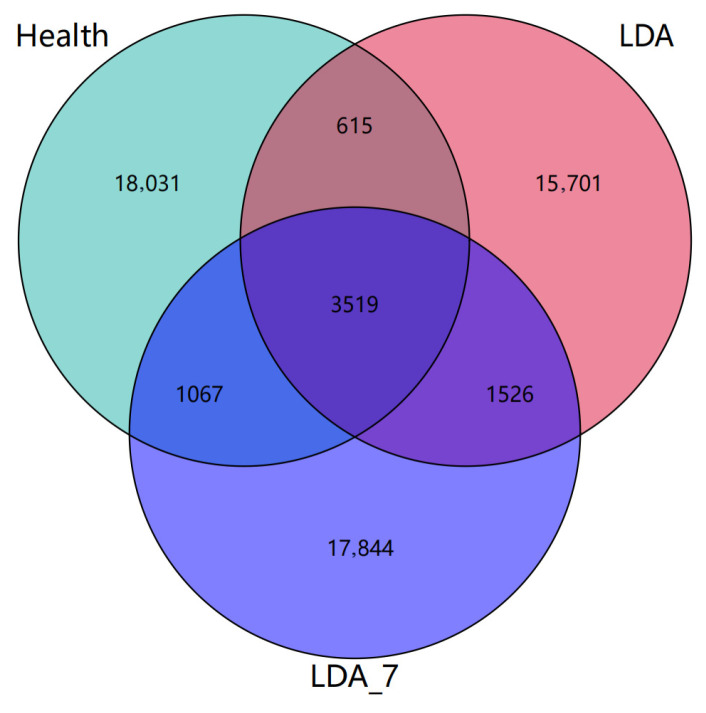
Venn diagram comparing the Operational Taxonomic Units (OTUs) of the rumen microbiota among the healthy, left-displaced abomasum (LDA) and cows with left-displaced abomasum 7 days after surgery (LDA_7) groups.

Venn diagrams were constructed to illustrate the unique and shared Operational Taxonomic Units (OTUs) among the three groups: the healthy group contained 18,031 unique OTUs (77.6% of the total OTUs) and 5201 shared OTUs (total: 23,231 OTUs), the samples from the LDA group contained 15,701 unique OTUs (73.5% of the total OTUs) and 5660 shared OTUs (total: 21,361 OTUs), and the samples from the LDA_7 group contained 17,844 unique OTUs (74.5% of the total OTUs) and 6112 shared OTUs (total: 23,956 OTUs). Compared with the healthy group and the LDA_7 group, the LDA group exhibited fewer OTUs (*p* < 0.05). Furthermore, compared with the LDA group, the healthy group shared a greater number of OTUs with the LDA_7 group (*p* < 0.05), indicating that these two groups exhibited greater similarity in certain aspects (Figure 1).

#### 3.1.2. Alpha Diversity Analysis

**Figure 2:**
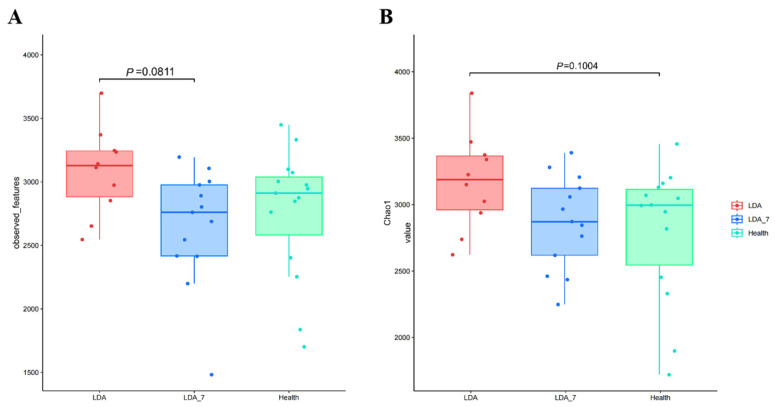
Alpha diversity analysis of the rumen microbiota among the healthy, left-displaced abomasum (LDA) and cows with left-displaced abomasum 7 days after surgery (LDA_7) groups by determination of the Chao1 (**A**) and observed feature (**B**) indices. The *X*-axis represents the group names, while the *Y*-axis represents the Alpha diversity indices.

Alpha diversity analysis revealed that both the Chao1 and observed feature indices remained at 300, with higher values in the LDA group than in the healthy group or the LDA_7 group, suggesting a greater abundance and diversity of rumen microbial communities in the LDA group (Figure 2).

#### 3.1.3. Beta Diversity Analysis

**Figure 3:**
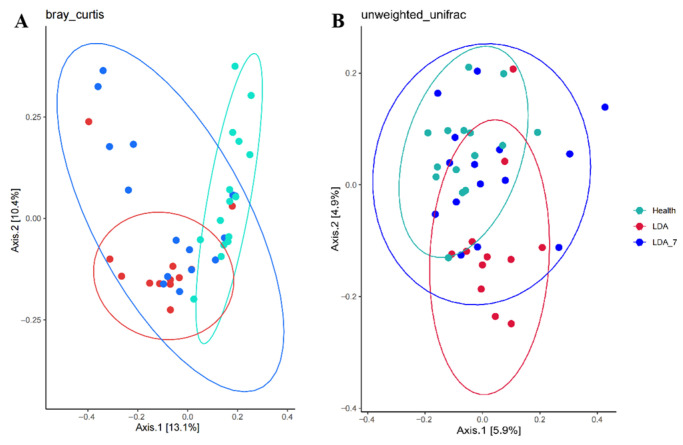
Beta diversity analysis of the rumen microbiota among the healthy, left-displaced abomasum (LDA) and cows with left-displaced abomasum 7 days after surgery (LDA_7) groups by PCoA based on the Bray–Curtis (**A**) and unweighted UniFrac (**B**) indices. LDA refers to left-displaced abomasum, LDA_7 refers to cows with left-displaced abomasum 7 days after surgery, and PCoA refers to Principal Coordinate Analysis. The horizontal axis (Axis 1) represents the first principal coordinate, with the percentage indicating the contribution of the first principal coordinate to the differences among samples. The vertical axis (Axis 2) represents the second principal coordinate, with the percentage indicating the contribution of the second principal coordinate to the differences among samples.

A comparative analysis of the ruminal microbial composition in the three groups was conducted using the Bray–Curtis and unweighted UniFrac distances with Principal Coordinate Analysis. The results of the Beta diversity analysis revealed that the samples from the LDA group and the healthy group formed tighter clusters, with some dispersion on the basis of the Bray–Curtis metric. Adonis analysis revealed a significant difference in community structure between healthy cows and cows with left-displaced abomasum (LDA) (R = 0.402, *p* < 0.01). Conversely, the samples from the LDA group and healthy group exhibited a less compact vertical distribution on the basis of the unweighted UniFrac metric. Adonis analysis showed a significant difference in community structure between healthy cows and LDA cows (R = 0.315, *p* < 0.01). The ruminal microbial structure in the LDA group differed significantly from that in the healthy group (*p* < 0.001, q = 0.003) (Figure 3, Table 2).

#### 3.1.4. LEfSe Analysis

**Figure 4:**
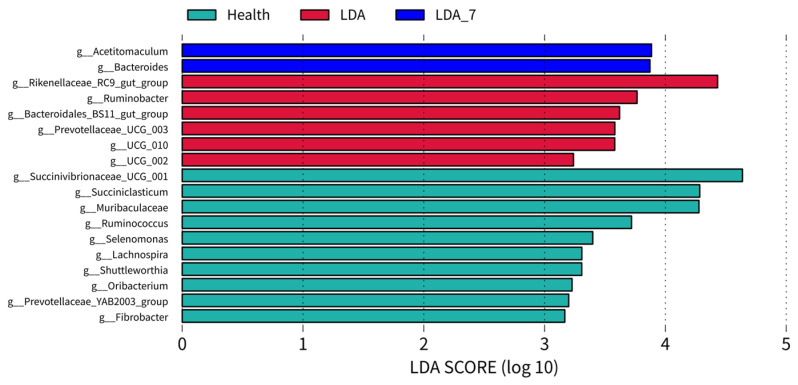
Linear Discriminant Analysis Effect Size (LEfSe) analysis of the differential rumen microbiota in genera between the healthy, left-displaced abomasum (LDA), and cows with left-displaced abomasum 7 days after surgery (LDA_7) groups.

Linear Discriminant Analysis Effect Size (LEfSe) analysis revealed 18 dominant microbial taxa at the genus level in the rumen microbiota of the three groups of dairy cows, with a linear discriminant analysis threshold of 3.0. There were 10 dominant microbial taxa in the healthy group, namely *Succinivibrionaceae_UCG_001*, *Succiniclasticum*, *Muribaculaceae*, *Ruminococcus*, *Selenomonas*, *Lachnospira*, *Shuttleworthia, Oribacterium*, *Prevotellaceae_YAB2003_group*, and *Fibrobacter*. There were six dominant microbial taxa in the LDA group, namely *Rikenellaceae_RC9_gut_group*, *Ruminobacter*, *Bacteroidales_BS11_gut_group*, *Prevotellaceae_UCG_003*, *Succinellaceae_UCG_010*, and *Succinellaceae_UCG_002*. There were two dominant genera in the LDA_7 group: *Acetitomaculum* and *Bacteroides* (Figure 4).

### 3.2. Structural Composition and Differential Analysis of the Bacterial Microbiota

#### 3.2.1. Differences at the Phylum Level

**Figure 5:**
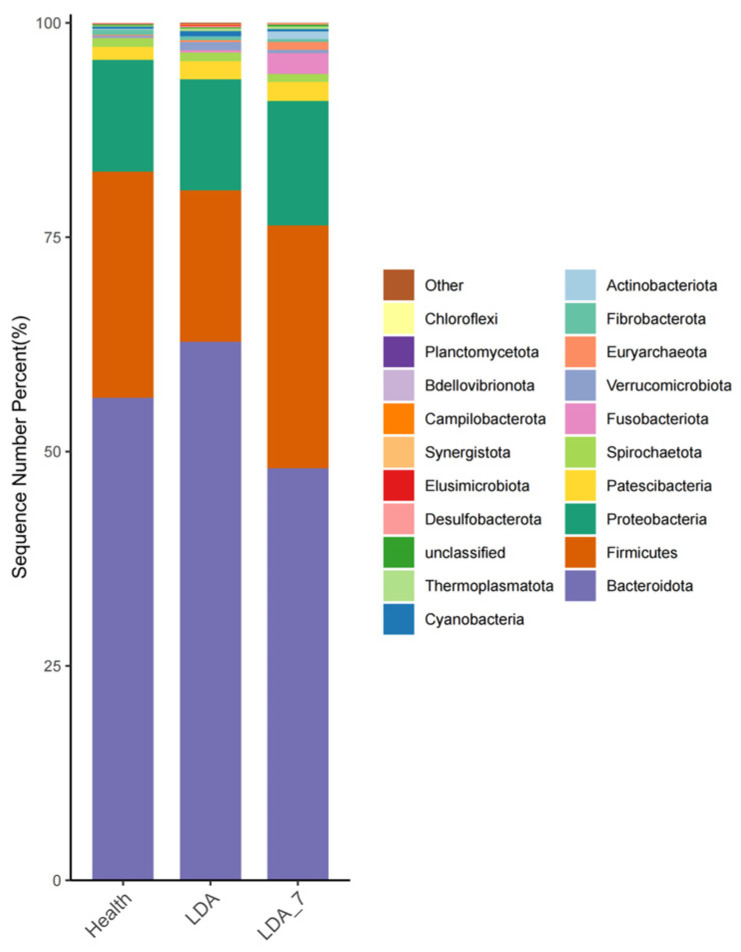
Relative abundances of rumen microbiota in phyla, top 20 between the healthy, left-displaced abomasum (LDA), and cows with left-displaced abomasum 7 days after surgery (LDA_7) groups.

At the phylum level, a total of 40 phyla were detected in the rumen microbiota across the three groups. The top 20 phyla, ranked by the relative abundance of rumen microorganisms, were as follows: *Bacteroidota*, *Firmicutes*, *Proteobacteria*, *Patescibacteria*, *Spirochaetota*, *Fusobacteriota*, *Verrucomicrobia*, *Euryarchaeota*, *Fibrobacterota*, *Actinobacteria*, *Cyanobacteria*, *Thermoplasmatota*, *Unclassified*, *Desulfobacterota*, *Elusimicrobiota*, *Synergistota*, *Campylobacterota*, *Bdellovibrionota*, *Planctomycetota*, and *Chloroflexota* (Figure 5).

**Figure 6:**
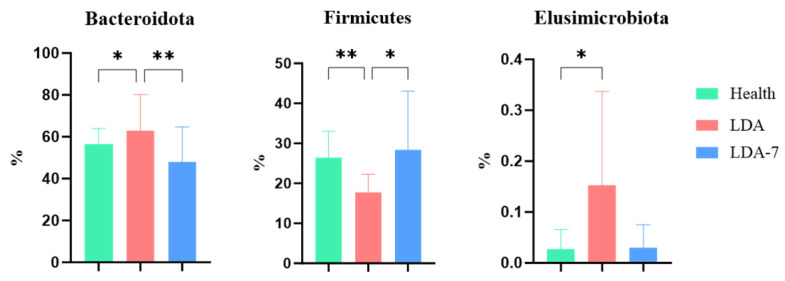
Comparison of rumen microbiota in phyla between the healthy, left-displaced abomasum (LDA), and cows with left-displaced abomasum 7 days after surgery (LDA_7) groups. The results are presented as “mean ± standard deviation”, * indicates *p* < 0.05 (significant difference), ** indicates *p* < 0.01.

The relative abundances of Bacteroidota and Elusimicrobiota in the rumen microbiota were significantly greater in the LDA group than in the healthy group (*p* < 0.05). The relative abundance of Bacteroidota was significantly greater in the LDA group than in the LDA_7 group (*p* < 0.01). The difference in the abundance of Elusimicrobiota between the LDA group and the LDA_7 group was not significant (*p* > 0.05). The relative abundance of Firmicutes was significantly lower in the LDA group than in the healthy group (*p* < 0.01). Additionally, the relative abundance of Firmicutes was significantly lower in the LDA group than in the LDA_7 group (*p* < 0.05, Figure 6).

#### 3.2.2. Differences at the Genus Level

**Figure 7:**
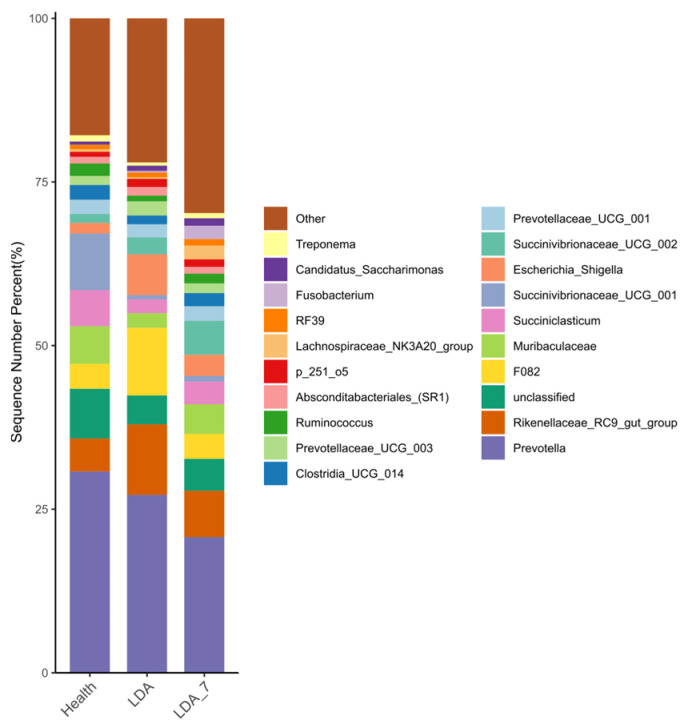
Relative abundances of rumen microbiota in genera, top 20 between the healthy, left-displaced abomasum (LDA), and cows with left-displaced abomasum 7 days after surgery (LDA_7) groups.

At the genus level, the analysis revealed the top 20 genera of rumen microorganisms ranked by their relative abundance, which are listed in order of their prevalence (Figure 7). These genera included *Prevotella*, *Rikenellaceae_RC9_gut_group*, *Unclassified*, *F082*, *Muribaculaceae*, *Succiniclasticum*, *Succinivibrionaceae_UCG_001*, *Escherichia_Shigella*, *Succinivibrionaceae_UCG_002*, *Prevotellaceae_UCG_001*, *Clostridia_UCG_014*, *Prevotellaceae_UCG_003*, *Ruminococcus*, *Absconditabacteriales_(SR1)*, *p_251_o5*, *Lachnospiraceae_NK3A20_group*, *RF39*, *Fusobacterium*, *Candidatus_Saccharimonas*, and *Treponema*.

**Figure 8:**
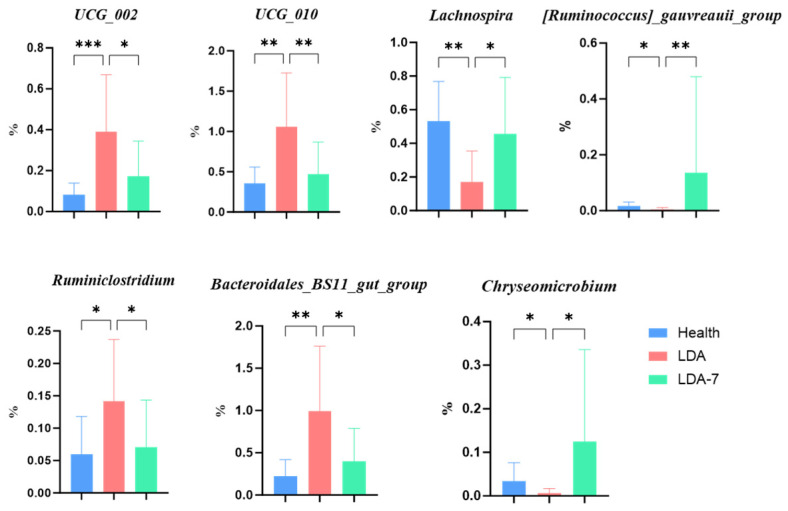
Genera with significant differences in the rumen microbiota among the healthy, left-displaced abomasum (LDA), and cows with left-displaced abomasum 7 days after surgery (LDA_7) groups. The results are presented as “mean ± standard deviation”, * indicates *p* < 0.05 (significant difference), ** indicates *p* < 0.01, *** indicates *p* < 0.001.

Compared with those in the healthy group, the abundances of *UCG_002*, *UCG_010*, and *Bacteroidales_BS11_gut_group* in the LDA group were significantly greater (*p* < 0.001, *p* < 0.01, and *p* < 0.01, respectively). Additionally, compared with that in the healthy group, the abundance of *Ruminiclostridium* in the LDA group was significantly greater (*p* < 0.05). In contrast, the abundance of *Lachnospira* in the LDA group was significantly lower than that in the healthy group (*p* < 0.01). Furthermore, the abundances of *[Ruminococcus]_gauvreauii_group* and *Chryseomicrobium* were significantly lower in the LDA group than in the healthy group (*p* < 0.05). When the LDA group was compared with the LDA_7 group, *UCG_010* showed an extremely significant increase in the LDA group (*p* < 0.01). Compared with the LDA_7 group, the LDA group also exhibited significantly increased abundances of *UCG_002*, *Bacteroidales_BS11_gut_group*, and *Ruminiclostridium* (*p* < 0.05). Conversely, the abundance of *[Ruminococcus]_gauvreauii_group* in the LDA group was significantly lower than that in the LDA_7 group (*p* < 0.01). Finally, compared with those in the LDA_7 group, the relative abundances of *Lachnospira* and *Chryseomicrobium* in the LDA group were significantly lower (*p* < 0.05) (Figure 8).

### 3.3. Predictive Analysis of Microbial Function

**Figure 9:**
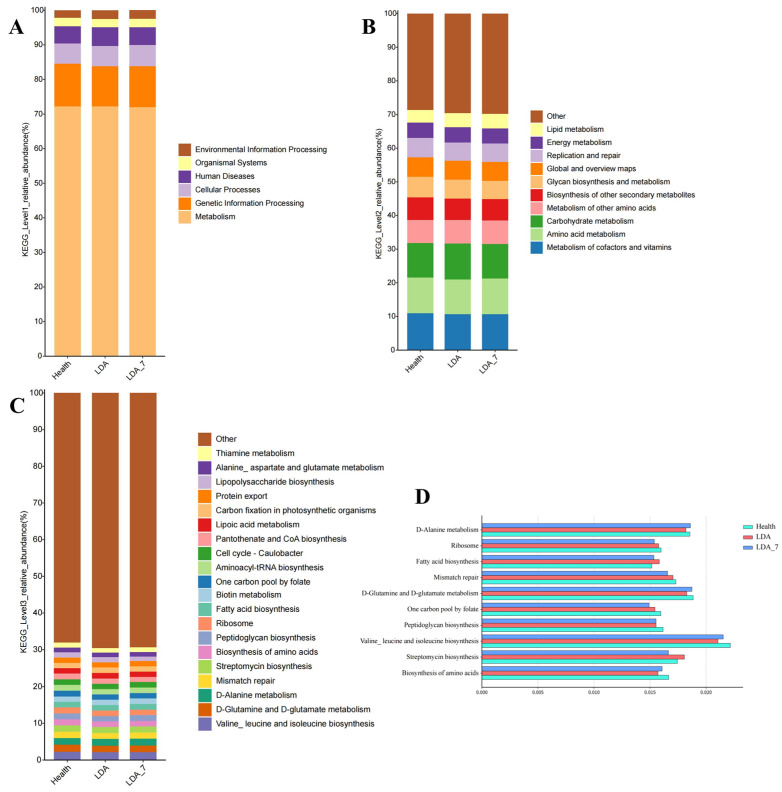
Bar charts of the predicted functional pathways of the dairy cow rumen microbiota based on the KEGG database. (**A**) KEGG_Level 1 pathways, (**B**) KEGG_Level 2 pathways, and (**C**) KEGG_Level 3 pathways. The vertical axis indicates the relative abundance percentage of the annotated functions, while the horizontal axis represents the group names. The functional categories corresponding to each color block are shown in the legend on the right. (**D**) Relative abundances of the top 10 KEGG Level 3 pathways among the healthy, left-displaced abomasum (LDA), and cows with left-displaced abomasum 7 days after surgery (LDA_7) groups.

Based on the functional pathway prediction results of rumen microbiota from the KEGG database, in the primary pathway, the rumen microbiota of dairy cows presented the greatest average relative enrichment in the metabolism category (72.34%). The remaining primary categories exhibited average relative enrichment in the following descending order: genetic information processing (12.1%), cellular processes (5.77%), human diseases (5.1%), organismal systems (2.46%), and environmental information processing (2.23%) (Figure 9A).

Forty-seven secondary pathways were predicted to be enriched. The top 10 in average relative abundance were cofactor and vitamin metabolism (10.76%), amino acid metabolism (10.66%), carbohydrate metabolism (10.27%), other amino acid metabolism (6.89%), biosynthesis of other secondary metabolites (6.63%), glycan biosynthesis and metabolism (5.94%), global and overview map (5.73%), replication and repair (5.62%), energy metabolism (4.55%), and lipid metabolism (4.06%). Nine of these pathways are related to metabolism, whereas one is associated with genetic information processing (Figure 9B).

A total of 380 tertiary pathways were predicted to be enriched. The top 10 pathways, ranked by their average relative enrichment, were biosynthesis of valine, leucine, and isoleucine (2.16%); D-glutamine and D-glutamate metabolism (1.86%); D-alanine metabolism (1.85%); streptavidin biosynthesis (1.73%); mismatch repair (1.70%); amino acid biosynthesis (1.62%); peptidoglycan biosynthesis (1.58%); ribosomes (1.57%); and the one-carbon pool by folate (1.54%) and fatty acid biosynthesis (1.54%) (Figure 9C,D).

#### Analysis of Differences in Tertiary Metabolic Functions

**Figure 10:**
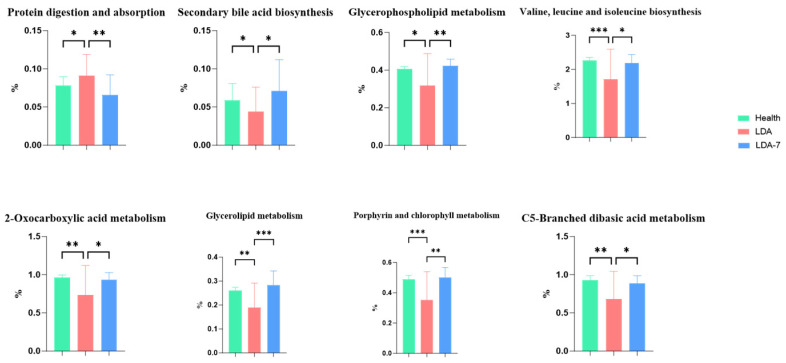
Comparative analysis of metabolic functions in tertiary pathways among the healthy, left-displaced abomasum (LDA), and cows with left-displaced abomasum 7 days after surgery (LDA_7) groups. The results are presented as “mean ± standard deviation”, * indicates *p* < 0.05 (significant difference), ** indicates *p* < 0.01, *** indicates *p* < 0.001.

Comparative analysis of the metabolic components of the tertiary pathways revealed that eight metabolic pathways were differentially enriched between the LDA group and the healthy and LDA_7 groups (Figure 10). Compared with those in the healthy group, the protein digestion and absorption pathways in the LDA group were significantly enriched (*p* = 0.0043). The following pathways exhibited significant underrepresentation in the LDA group: valine, leucine, and isoleucine biosynthesis; 2-oxocarboxylic acid metabolism; porphyrin and chlorophyll metabolism; and C5-branched dibasic acid metabolism (*p* = 0.0009, *p* = 0.007, *p* = 0.0003, and *p* = 0.0013, respectively). Secondary bile acid biosynthesis and glycerophospholipid metabolism pathways were also significantly underrepresented in the LDA group compared with the healthy group (*p* = 0.0495, *p* = 0.0172). The protein digestion and absorption pathway was highly significantly enriched in the LDA group compared with the LDA_7 group (*p* = 0.0043). Compared with those in the LDA_7 group, the porphyrin and chlorophyll metabolism and glycerolipid metabolism pathways in the LDA group were significantly underrepresented (*p* = 0.0017 and *p* = 0.0041, respectively). The following pathways also exhibited significant underrepresentation in the LDA group compared with the LDA_7 group: valine, leucine, and isoleucine biosynthesis; 2-oxocarboxylic acid metabolism; C5-branched dibasic acid metabolism; glycerophospholipid metabolism; and secondary bile acid biosynthesis (*p* = 0.0228, *p* = 0.039, *p* = 0.0349, *p* = 0.0041, *p* = 0.0205, respectively).

### 3.4. Correlations Between Genera and Metabolic Pathways

**Figure 11:**
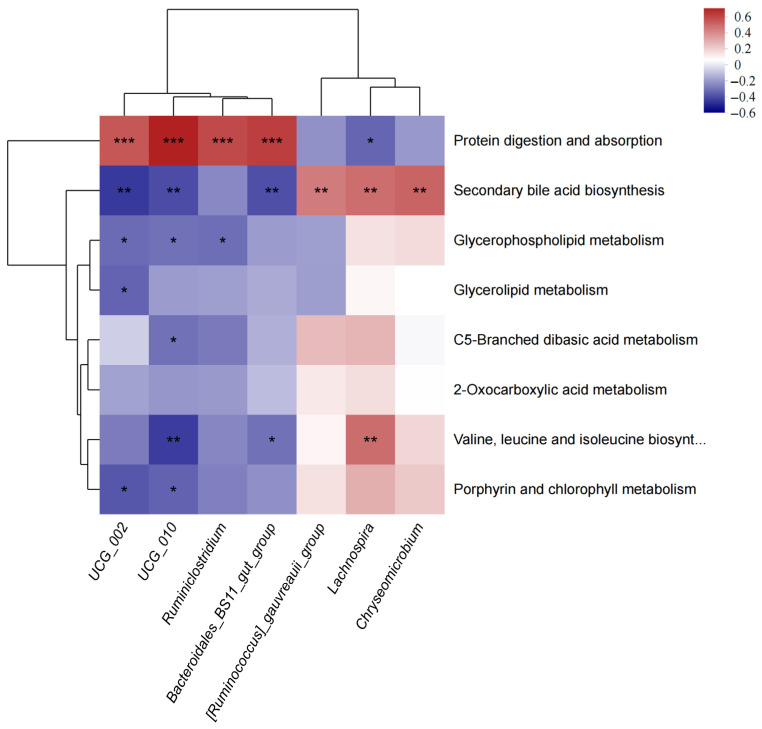
Correlation analysis between metabolic pathways and genera in the rumen microbiota. The *X*-axis represents the differential bacterial genera, while the *Y*-axis represents the differential metabolic pathways. The R and *p* values were obtained through Spearman correlation analysis; * indicates *p* < 0.05 (significant difference), ** indicates *p* < 0.01, *** indicates *p* < 0.001.

Spearman correlation analysis was performed between bacterial species and metabolic pathways, and the results revealed that there were correlations between different bacterial species and different metabolic pathways (Figure 11). Positive correlations were observed between *UCG_010* and protein digestion and absorption (R = 0.067) and between *Bacteroidales_BS11_gut_group* and protein digestion and absorption (R = 0.592). Conversely, negative correlations were seen between *UCG_010* and valine, leucine and isoleucine biosynthesis (R = −0.449) and *UCG_002* and secondary bile acid biosynthesis (R = −0.449).

## 4. Discussion

Left-displaced abomasum (LDA) is a type of digestive tract obstruction disease, characterized by the displacement of the true stomach from the right side of the abdominal cavity to the left side through the bottom of the rumen [35]. The rumen is a specialized digestive organ unique to ruminants, and the rumen microbiota plays a crucial role in it by effectively degrading cellulose and thus ensuring adequate nutritional intake for ruminants [36]. With the increase in the proportion of concentrate in the diet, the abundance and diversity of the microbial community tend to decline. Feeding a high-concentrate diet can indeed meet the nutritional requirements of livestock to some extent [37]. However, long-term consumption of such a diet can lead to an imbalance in the gastrointestinal (GI) environment of dairy cows, causing damage to the GI structure and consequently affecting its normal function. In more severe cases, it may even induce nutritional metabolic diseases, which can negatively impact the GI barrier, resulting in the inability of cows to absorb nutrients from feed and potentially triggering LDA. The composition of the rumen microbiota is not only associated with the digestive efficiency of dairy cows but also has a certain connection with their production performance. Low-efficiency cows have a higher proportion of *Succinivibrio* and *Butyricimonas*, while high-efficiency cows have a higher abundance of *Faecalibacterium* in the total rumen bacterial community. When studying the rumen microbiota of high-yielding dairy cows, Mu found that the bacterial richness and evenness were lower than those of low-yielding cows [36]. Additionally, ruminal microorganisms perform various other functions, including the decomposition and synthesis of sugars and proteins [36].

In this study, we employed 16S rRNA sequencing to compare the rumen microbiota in the healthy group, the left-displaced abomasum (LDA) group, and the cows with left-displaced abomasum 7 d after surgical treatment (LDA_7) group. The results indicated increases in the relative abundance of four genera, including *UCG_002*, which were consistent with changes in protein digestion and absorption. The relative abundances of *Lachnospira* and three other *Firmicutes* genera decreased in the rumen microbiota in the LDA group, mirroring the changes observed in seven metabolic pathways. The results indicated that the occurrence of LDA would lead to an increase in the relative abundance of four genera in the rumen microbiota of ruminants, namely *UCG-002*, *UCG-010, Bacteroidales_BS11_gut_group*, and *Ruminiclostridium*, and would affect protein digestion and absorption. This finding is consistent with the study by Song [36], which reported that the relative abundance of certain microbial taxa, such as *UCG-002* and *UCG-010*, was significantly higher in cows with LDA compared to healthy cows, and these taxa were associated with changes in protein digestion and absorption pathways. Additionally, the study by Xue [38] also found that the relative abundance of *Bacteroidales_BS11_gut_group* was increased in the rumen microbiota of cows with LDA, which might be related to the alterations in rumen metabolic functions.

The dominant genera in the LDA group included *Rikenellace_RC9_gut_group*, *Ruminococcus*, *Bacteroidales_BS11_gut_group*, *Prevotellaceae_UCG_003*, *UCG_010*, and *UCG_002*. In contrast, the predominant genus in the healthy group was *Fibrobacter*, which is the only anaerobic Gram-negative bacterium within the *Cellulobacteriaceae* family of the order *Cellulobacteriales* and typically ferments cellulose and cellobiose [39,40]. The dominance of *Fibrobacter* in the healthy group highlights its critical role in cellulose fermentation, producing key volatile fatty acids such as acetic and succinic acids, which are essential for rumen health and energy supply to the host [27,36]. The abundance of *Ruminococcus* is influenced by a high-concentrate diet; the high abundance of *Ruminococcus* in the LDA group, influenced by a high-concentrate diet, aligns with previous findings that link this genus to ruminal acidosis, a condition often observed in cows with LDA [18]. Additionally, Guardia’s study indicated that the high relative abundance of *Succinivibrionaceae_UCG_001* in the healthy group and its positive correlation with milk production suggest that this genus may contribute to improved feed efficiency and overall performance in dairy cows [41,42]. These findings underscore the complex interplay between the rumen microbiota and host health, highlighting the potential for microbial interventions to enhance rumen function and mitigate metabolic disorders such as LDA.

In the microbiota analysis, the phylum and genus levels served as critical markers, providing essential taxonomic information about the microbiota [18,43]. This analysis also revealed insights into the composition, similarity, and relationships of the microbiota. Bacteroidota was identified as the dominant phylum, with the highest average relative abundance (55.2%). The relative abundance of Bacteroidota in the LDA group significantly exceeded that in both the healthy group and the LDA_7 group, while the relative abundance of Firmicutes was significantly lower in the LDA group than in the healthy group or the LDA_7 group. These findings aligned with the results of most rumen metagenomic studies that identified Bacteroidota and Firmicutes as the dominant phyla [44,45]. The dominance of Bacteroidota in the microbiota is consistent with findings from various studies indicating its significant role in rumen fermentation and overall microbiome function [45]. Bacteroidota, particularly the genus Bacteroides, is known for its ability to degrade complex carbohydrates, contributing to the production of short-chain fatty acids (SCFAs) such as acetate and succinate, which are crucial for ruminant nutrition [46]. The relatively high abundance of Firmicutes and Proteobacteria further highlights the complexity and functional diversity of the rumen microbiota. These phyla are often associated with a range of metabolic activities, including the breakdown of plant cell walls and the fermentation of various substrates [45]. This evidence further confirmed the critical role of these two phyla in the rumen microbial community. The LDA group exhibited reduced food intake, severely diminished appetite, and decreased dietary consumption. These conditions resulted in alterations to the microbial structure within the rumen of dairy cows, which is consistent with the findings of Mao and Hua [18,47].

The average relative abundance of 16 genera, including *Prevotella*, exceeded 1%, indicating the high abundance of these genera. The high abundance of *Prevotella* in the rumen microbiota is consistent with previous studies indicating its significant role in rumen fermentation and overall microbiome function [48]. *Prevotella* is known for its ability to degrade complex carbohydrates and proteins, contributing to the production of short-chain fatty acids (SCFAs) such as acetate and succinate, which are crucial for ruminant nutrition [48]. The presence of *Prevotella* as a dominant genus underscores its importance in the rumen ecosystem, where it plays a key role in both protein degradation and starch utilization.

The average relative abundance of 87 genera, such as *RF39*, ranged from 0.1~1%, indicating that they were low-abundance genera. The average relative abundance of the remaining genera was less than 0.1%, indicating that they were ultralow-abundance genera. No significant differences were observed among the three groups because these genera constitute the core microbiota in the digestive tract of dairy cows, resulting in their relatively stable abundance among rumen bacteria. This stability aligns with the concept of a “core microbiome”, which refers to a set of microbial taxa that are consistently present across different individuals and environmental conditions [49]. The presence of a stable core microbiota suggests that certain microbial functions are conserved across different conditions, providing a baseline for rumen function that is less influenced by external factors such as diet or physiological state [50,51].

The rumen microbiota endows the host with fundamental functions that facilitate the breakdown of food into smaller, more digestible molecules, enabling the animal to extract more energy from its diet [50,52,53]. Amino acids serve as essential nutrients for the growth and metabolism of rumen microorganisms [54]. Our study revealed a negative correlation between UCG_010 and the biosynthesis of valine, leucine, and isoleucine. Additionally, the abundance of UCG_010 was significantly elevated in dairy cows suffering from left-displaced abomasum. The activity of valine, leucine, and isoleucine biosynthesis in the LDA group was significantly lower than that in the healthy group. Valine, leucine, and isoleucine are branched-chain amino acids and are the only amino acids metabolized outside the liver. They account for approximately 35% of the essential amino acids in skeletal muscle proteins and serve as the primary amino acids for energy supply in the body [55]. These findings indicate that LDA severely affects the health of dairy cows and suggest that amino acids could be judiciously added to feed to improve the nutritional status and production performance of dairy cows. The LDA group exhibited significantly lower triglyceride metabolism activity. Consequently, regulating triglyceride metabolism and controlling triglyceride concentrations are essential for effective rumen health management.

Normal microbiota regulate the balance of the microecological environment, allowing microorganisms, animals, and the external environment to maintain their stability and enabling the body to achieve an optimal state [47]. Consequently, nutrients are fully utilized, ensuring optimal production performance and rapid quality enhancement. As our results show, the secondary bile acid biosynthesis pathway was negatively correlated with *UCG_002*. While fatty acids increase the synthesis of bile acids and promote the conversion of primary bile acids to secondary bile acids [54]. Protein digestion and absorption are two distinct yet closely linked processes, with digestion occurring primarily in the abomasum and small intestine. The digestive system breaks down proteins by secreting pepsin, whose activity produces readily digestible small peptides and amino acids. In the small intestine, trypsin, along with other enzymes secreted by small intestinal glands, continues to digest these polypeptides and amino acids, breaking them down into even smaller molecules that can be absorbed and utilized by the body [55]. Future studies should confirm these interactions in vitro.

In conclusion, the relative abundances of *UCG_002*, *UCG_010*, *Ruminiclostridium*, and *Bacteroidales_BS11_gut_group* decreased but that of *Lachnospira* increased in the LDA_7 group. Compared with the healthy group, the LDA_7 group did not significantly differ, indicating that the microbial abundance returned to a healthy level. The *UCG_002*, *UCG_010*, and *Ruminiclostridium* genera belong to the phylum Firmicutes, whereas the *Bacteroidales_BS11_gut_group* belongs to the phylum Bacteroidota. Zhou’s study demonstrated that *Firmicutes* and *Bacteroidota* were the dominant phyla in the rumen of dairy cows [56]. Firmicutes primarily decompose fiber, while Bacteroidota mainly decomposes nonfiber carbohydrates. Additionally, the percentage of milk fat was strongly positively correlated with the proportions of Firmicutes and Bacteroidota [38,57]. Although direct evidence linking changes in the abovementioned genera to LDA is lacking, it is plausible that an imbalance in the intestinal microbiota in dairy cows could disrupt normal rumen fermentation and nutrient absorption, potentially leading to metabolic disorders and increasing the risk of LDA. Future studies should be conducted to validate these interactions in vitro.

## 5. Conclusions

The results of this experiment demonstrated that the rumen microbiota in the LDA group exhibited elevated species richness, diversity, and complexity. 16S rRNA sequencing revealed seven differentially abundant taxa between the LDA group and the healthy group, namely *UCG_002*, *UCG_010*, *Bacteroidales_BS11_gut_group*, *Ruminiclostridium*, *Lachnospira*, *Chryseomicrobium*, and *Firmicutes*. Following treatment, the abundances of these seven taxa returned to those observed in healthy cows. These taxa play significant roles in regulating host protein absorption and digestion as well as protein digestion and absorption. These taxa could also serve as potential early warning markers for the detection of LDA in dairy cows or as new targets for the development of effective prevention strategies.

## Figures and Tables

**Table 1:** Litter size, monthly age, and days of lactation of experimental cows.

Group	Age	Litter Size	Days of Lactation
Health	2.87 ± 1.13	2.00 ± 1.13	17.07 ± 3.53
LDA	3.73 ± 1.67	2.20 ± 1.32	17.27 ± 4.45

**Table 2:** Permutational Multivariate Analysis of Variance (PERMANOVA) similarity analysis based on Bray–Curtis and unweighted Unifrac distance metrics.

	Group1	Group2	Pseudo-F	*p* Value	q-Value
Bray–Curtis	LDA	Health	3.700	0.001	0.002
	LDA	LDA_7	1.238	0.130	0.130
	LDA_7	Health	2.398	0.001	0.002
Unweighted Unifrac	LDA	Health	1.583	0.001	0.003
	LDA	LDA_7	1.127	0.090	0.090
	LDA_7	Health	1.217	0.020	0.030

## Data Availability

Data are contained within this article, and further inquiries can be directed to the corresponding authors.
